# Navigating the Global Regulatory Landscape for Exosome-Based Therapeutics: Challenges, Strategies, and Future Directions

**DOI:** 10.3390/pharmaceutics17080990

**Published:** 2025-07-30

**Authors:** Nagendra Verma, Swati Arora

**Affiliations:** 1 Applied Education in the MedTech Industry, St. Cloud State University, Plymouth, MN 55442, USA; 2 Department of Chemistry, University of Pittsburgh, 219 Parkman Ave, Pittsburgh, PA 15260, USA; swa10@pitt.edu

**Keywords:** exosomes, extracellular vesicles, therapeutics, clinical translation, regulatory framework, quality control, standardization, global regulatory harmonization, manufacturing scalability

## Abstract

Extracellular vesicle (EV)-based therapies have attracted considerable attention as a novel class of biologics with broad clinical potential. However, their clinical translation is impeded by the fragmented and rapidly evolving regulatory landscape, with significant disparities between the United States, European Union, and key Asian jurisdictions. In this review, we systematically analyze regional guidelines and strategic frameworks governing EV therapeutics, emphasizing critical hurdles in quality control, safety evaluation, and efficacy demonstration. We further explore the implications of EVs’ heterogeneity on product characterization and the emerging direct-to-consumer market for EVs and secretome preparations. Drawing on these insights, in this review, we aim to provide a roadmap for harmonizing regulatory requirements, advancing standardized analytical approaches, and fostering ongoing collaboration among regulatory authorities, industry stakeholders, and academic investigators. Such coordinated efforts are essential to safeguard patient welfare, ensure product consistency, and accelerate the responsible integration of EV-based interventions into clinical practice.

## 1. Introduction

Exosomes are nanoscale extracellular vesicles (EVs), around 30–100 nm, released by diverse cell types, facilitating intercellular communication via the transfer of biomolecules [1,2,3]. Moreover, they are hypothesized to carry specific biomarkers reflective of their parent cells, thereby offering significant insights into disease states and potential therapeutic strategies [4,5]. Common markers used for exosome characterization include tetraspanins (CD9, CD63, CD81), Alix, TSG101, and heat shock proteins [6]. Their roles in immune modulation, cell survival, and angiogenesis further underscore their importance in both physiological regulation and pathological conditions [1,7]. For instance, stem cell-derived exosomes have demonstrated the capacity to enhance tissue repair and regeneration through the modulation of inflammation and promotion of angiogenesis, which may benefit conditions such as diabetic complications and neurodegenerative disorders [8,9]. Furthermore, the natural capacity of exosomes to serve as drug carriers offers a biocompatible and targeted delivery system for therapeutics [10,11].

Emerging applications in therapeutics, diagnostics, and drug delivery have brought exosomes to the forefront of biomedical research. In the therapeutic sphere, mesenchymal stem cell-derived exosomes are being actively investigated for their regenerative properties, especially in treating osteoarthritis and diabetic wounds [9,12]. Additionally, exosome-based immunotherapy is showing promise in cancer treatment, as it facilitates the targeted delivery of immunotherapeutics while minimizing systemic toxicity [13,14]. As diagnostic tools, exosomes are considered promising candidates for liquid biopsies because they can mirror the pathological state of their originating tissue, thereby aiding in the early and non-invasive detection of cancers and other diseases [4,5]. Consequently, the potential for personalized medicine through tailored exosome therapies emphasizes the need for further research alongside careful ethical considerations.

The increased interest in both clinical translation and commercialization of exosome-based products is accompanied by several challenges. Regulatory bodies are anticipated to develop comprehensive guidelines addressing the characterization, safety, and efficacy of these products, which is vital for mitigating concerns related to immunogenicity and long-term effects [12,15]. Equally, preclinical and clinical trials are essential to validate therapeutic benefits and standardize production methods [2,15]. Challenges remain in managing EV source variability, scaling up production, standardizing isolation and characterization protocols, and ensuring batch-to-batch consistency regarding safety and efficacy [5]. Institutions like the Food and Drug Administration (FDA) and the European Medicines Agency (EMA) have created frameworks highlighting the significance of adhering to good manufacturing practices and robust quality control standards. Concurrently, initiatives to commercialize products aim to improve the production yield, purity, and stability of EV formulations [5,6,16,17,18,19,20,21,22]. This robust growth trajectory in clinical development suggests that EV may soon play a central role in advancing precision medicine across diagnostics, therapeutics, and drug delivery [5].

On a regional level, regulatory agencies have begun formulating guidelines specific to EV-based products; however, discrepancies in classification and evaluation criteria still exist across jurisdictions. For example, while the FDA regulates EV under the Public Health Service Act, the EMA may classify certain EV therapies as advanced therapy medicinal products (ATMPs) depending on their content and function [23]. Similarly, countries such as South Korea, Japan, and Taiwan have either implemented or are in the process of developing distinct regulatory strategies that reflect local scientific priorities and healthcare needs [23]. This situation underscores the necessity for global regulatory harmonization aimed at streamlining clinical translation and commercialization processes while ensuring public health safety [24]. Ultimately, establishing a unified global framework is crucial to maintain consistent manufacturing practices, protect patient safety, and facilitate the responsible translation of EV research into clinical applications. Collaboration among regulatory agencies, researchers, and industry stakeholders will be key to fostering an innovative yet secure regulatory environment. Moreover, in this review, we provide a comprehensive analysis of the current global regulatory framework, challenges, compare regional differences, and propose strategies for achieving global harmonization.

## 2. Exosome-Based Therapeutics in Clinical Trials

Exosome-based therapeutics have attracted considerable scholarly attention in recent years, as illustrated by the growing number of clinical trials conducted across diverse medical fields. Their potential is being investigated for an array of conditions, including cancer, cardiovascular diseases, neurodegenerative disorders, and inflammatory ailments. In particular, considerable focus has been placed on mesenchymal stem cell (MSC)-derived exosomes due to their regenerative and immunomodulatory capabilities. For instance, clinical trials evaluating their application in COVID-19 pneumonia have provided evidence that these exosomes can expedite recovery through anti-inflammatory and reparative mechanisms [25,26,27]. Moreover, a clinical trial employing nebulization therapy with MSC-derived exosomes has been initiated to assess their benefits in treating severe pulmonary complications associated with SARS-CoV-2 infections [25]. Similarly, studies examining MSC-derived exosomes for premature ovarian insufficiency (POI) have indicated promising therapeutic potential in reproductive health [28,29].

In the field of oncology, exosomes are under active investigation as vehicles for targeted drug delivery, particularly in RNA-based therapeutic applications. These vesicles may enhance treatment protocols and serve as carriers for interfering RNAs (siRNAs) and messenger RNAs (mRNAs) [30,31]. Contemporary clinical trials in pancreatic cancer and glioblastoma are evaluating exosome-based drug delivery systems that aim to increase cytotoxicity against tumors while reducing systemic toxicity [32]. Furthermore, exosome-based cancer vaccines are being explored, capitalizing on the inherent ability of these vesicles to stimulate immune responses against specific malignancies [10,33]. In addition, subsequent studies have broadened the scope of exosome applications; for example, in the context of neuroprotection following ischemic strokes, several clinical trials suggest that exosomes may facilitate recovery through neuroplastic mechanisms [34,35]. Preliminary evidence in gastrointestinal diseases further indicates that exosome-based therapies may modulate immune responses and promote tissue repair in conditions such as inflammatory bowel disease [5,36].

Despite these encouraging advancements, the translation of exosome research into clinical applications faces numerous regulatory challenges. The intrinsic complexity of exosomes as biological entities mandates the establishment of stringent frameworks to ensure their safe and effective use as therapeutic agents. Accordingly, the following discussion examines the principal regulatory challenges currently confronting exosome research, particularly in the context of clinical trials and the development of exosome-based therapies.

First, a prominent challenge pertains to the classification of EVs within existing regulatory frameworks. As EVs may be categorized as either biological products or drug delivery systems, their regulatory oversight becomes inherently complex [9,37]. Considering that EV therapies are relatively novel, current regulatory guidelines may not fully address the unique characteristics and functionalities of these vesicles. Regulatory agencies, such as the U.S. FDA, are still in the process of developing the requisite policies to govern the production, clinical trials, and therapeutic applications of EV [9,15]. Furthermore, the absence of universally accepted protocols for exosome isolation and characterization further compounds these regulatory hurdles [2,37].

Moreover, the regulatory approval process demands an in-depth understanding of both the pharmacokinetics and therapeutic efficacy of EV therapies [15]. This process necessitates rigorous preclinical and clinical evaluations to establish safety and effectiveness, frequently involving extensive data collection regarding the behavior and effects of exosomes in varied biological environments. The challenge is further compounded by the need for researchers to delineate the intricate pathways of exosome biogenesis, uptake, and functionality to provide compelling evidence for regulatory submissions [15]. As highlighted in reviews focusing on exosome dynamics, these factors must be systematically characterized to facilitate the seamless translation of exosome-based therapies into clinical practice [37,38].

In addition, issues related to the standardization of exosome preparations continue to impede their clinical application. Given that individual studies often employ different methods for exosome isolation, variability in exosomal composition and functional outcomes is observed [5]. Consequently, regulatory agencies face significant difficulties in evaluating the consistency and reliability of EV-based products [2,39]. The lack of standardized quality controls may lead to substantial variances in therapeutic outcomes, thereby posing a barrier to the recognition of EV-based interventions as established clinical therapies.

Further complicating the regulatory landscape is the dynamic nature of EV content, which can be influenced by factors such as cell type, disease state, and environmental conditions [40]. Accordingly, regulatory frameworks must be sufficiently adaptable to accommodate these variables and accurately capture the biological intricacies of exosomes as therapeutic agents. This evolving scenario necessitates continuous dialogue between researchers and regulatory authorities to formulate guidelines that foster innovation while ensuring patient safety [2,41].

**Figure 1:**
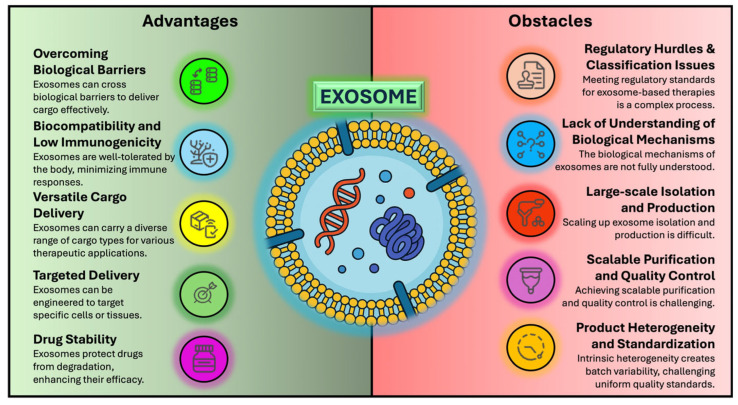
Comparative overview of the principal advantages and key challenges in exosome-based therapeutics. (**Left Panel**): core advantages—efficient barrier crossing, high biocompatibility and low immunogenicity, versatile cargo loading, targeted delivery, and improved stability. (**Right Panel**): main hurdles—regulatory ambiguity, mechanistic gaps, scale-up and purification constraints, quality control issues, and batch heterogeneity.

In summary, although EV research holds considerable promise for advancing therapeutic strategies, it is encumbered by regulatory challenges arising from classification uncertainties, the imperative for robust pharmacological data, and the complexities of standardization. Overcoming these obstacles will be pivotal in the successful integration of EV-based therapies into clinical practice, an endeavor that will require concerted collaborative efforts among stakeholders in the biomedical field [15,37,38] (Figure 1).

## 3. Global Regulatory Frameworks for EV

The global regulatory frameworks that govern the development and approval of EV-based therapies are both intricate and regionally disparate (Table 1), thereby influencing their clinical translation. Consequently, exosomes—as biologic medicines—face significant regulatory hurdles owing to their unique intracellular mechanisms and heterogeneous manufacturing techniques, which impede efforts at standardization. Globally, regulation generally hinges on two primary strategies: firstly, evaluation of the molecular and physiological effects of EV cargo; and secondly, assessment based on the methods of EV acquisition and production [15]. Under the first strategy, regulators determine whether bioactive components—such as functional RNAs or modified proteins—elicit therapeutic or diagnostic effects, thus classifying the product as a biological drug or an ATMP [15]. In contrast, the second approach—common in certain Asian jurisdictions—categorizes EV-based products according to their provenance (e.g., isolation from living cells, bioengineering, or derivation from nonliving materials), irrespective of cargo composition [15]. Furthermore, EV-based therapies are typically regulated akin to biological medicinal products, requiring in-depth characterization of molecular composition, structure, pharmacokinetics, and therapeutic efficacy, although these requirements continue to challenge regulatory agencies [15].

Regional disparities further shape approval pathways (Table 1). For instance, in Europe, EVs are not considered ATMPs unless incorporated into gene therapies, a classification that directly affects regulatory strategy, jurisdictional oversight, and development timelines [42]. Although the EU’s ATMP Regulation provides a harmonized framework for regenerative medicine, it has been criticized for not keeping pace with rapid technological advances [43]. In the United States, regulatory responsibilities are shared between the Center for Biologics Evaluation and Research (CBER) and the Center for Drug Evaluation and Research (CDER), with oversight determined by the specific application of the EV product [42]. Meanwhile, Japan’s Pharmaceuticals and Medical Devices (PMD) Act and the Act on the Safety of Regenerative Medicine (ASRM) permit conditional, time-limited marketing authorizations, thereby expediting development while safeguarding patient welfare [44]. The International Society for Extracellular Vesicles (ISEV) emphasizes the necessity of collaboration among researchers, clinicians, and regulatory authorities to ensure the safe and effective clinical translation of EV-based therapies [45]. Despite the expanding promise of EVs in regenerative medicine, oncology, and targeted drug delivery, critical obstacles—particularly those relating to isolation, purification, and regulatory standardization—remain [2]. Overall, while significant progress has been made, regulatory frameworks must evolve further to address the unique challenges posed by EV-based therapies and to facilitate their clinical application [46,47].

Overall, significant progress has been made in recognizing the potential of EV-based therapies, yet numerous challenges remain in their regulatory oversight. The development of comprehensive guidelines addressing the isolation, characterization, and therapeutic efficacy of EV, along with international regulatory harmonization, will be crucial in enabling these innovative therapies to fulfill their promise in clinical settings.

On the other hand, regulatory frameworks for bioengineered EVs and exosome-based therapeutics are still evolving but currently hinge on established biologics and advanced therapy medicinal product (ATMP) pathways. Initially, regulatory agencies such as the FDA and EMA treated naïve EV-based therapeutics under existing biologics or ATMP frameworks—requiring demonstration of identity, purity, potency, and safety analogous to protein biologics and cell therapies; however, with the advent of bioengineered EVs (encompassing cargo-loaded vesicles and surface-functionalized constructs), regulators are beginning to adopt risk-based classification schemes that accommodate these modifications without reinventing guidance entirely [48]. Consequently, engineered EVs may be regulated variably as biologics, combination products, or gene-therapy medicinal products depending on their composition and intended function, thereby invoking combination-product pathways under Section 351 of the U.S. Public Health Service Act or Regulation 1394/2007 in the EU [15]. In the United States, the FDA treats engineered EVs as biological products under the Public Health Service Act, requiring Investigational New Drug (IND) applications, detailed chemistry manufacturing control (CMC) documentation, potency assays, and rigorous viral safety testing prior to clinical trials [15,49]. In Europe, the EMA classifies exosome preparations as ATMPs under Regulation (EC) No 1394/2007, mandating Good Manufacturing Practice (GMP) compliance, comprehensive source material characterization, nonclinical safety studies, and clinical trial authorization through the Committee for Advanced Therapies (CAT) process [50]. Moreover, CMC requirements now mandate validation of cargo encapsulation efficiency, detailed characterization of surface modifications, and lot-to-lot consistency through advanced analytics such as single-vesicle profiling. Furthermore, preclinical safety assessments and clinical trial designs must reflect altered biodistribution and immunogenicity profiles of engineered EVs, driving the integration of adaptive safety-monitoring plans and comprehensive pharmacovigilance strategies that track both EV-related and cargo-related adverse events. At the global level, ICH and WHO have initiated harmonization efforts—such as ICH Q5E on biotechnology-derived proteins and WHO draft guidelines on vesicle therapeutics—to align nomenclature, quality standards, and preclinical models, although regional differences in classification criteria and documentation requirements persist and international harmonization remains incomplete, highlighting the urgent need for a unified, risk-based framework that explicitly addresses both naïve and engineered EV products to accelerate safe global translation [15,51,52].

In summary, naïve extracellular vesicles have been regulated under existing biologics and ATMP frameworks, with emphasis on particle characterization, purity, potency and safety. In contrast, bioengineered EVs now fall under risk-based schemes that add requirements for validating cargo loading, surface modifications and batch consistency. While the FDA and EMA continue to use IND and ATMP pathways, global harmonization is still emerging. From the author’s perspective, a unified, risk-based regulatory framework that explicitly covers both naïve and engineered vesicles—without diluting the rigor of current biologics oversight—will be essential to ensure their safe and effective clinical translation.

### 3.1. United States Regulatory Framework

In the United States, products based on EV for treating or preventing diseases are categorized as drugs and biologics, falling under the regulatory frameworks of the Public Health Service (PHS) Act Section 351 and the Federal Food, Drug, and Cosmetic (FD&C) Act [15,52]. Accordingly, sponsors are required to submit an Investigational New Drug (IND) application and, following successful clinical trials, file a Biologics License Application (BLA) with the FDA’s Center for Biologics Evaluation and Research (CBER) [52]. Furthermore, the FDA has consistently issued public safety notices and undertaken enforcement actions against the marketing of unapproved EV products, thereby highlighting regulatory gaps and the urgent need for comprehensive guidelines [15]. To ensure patient safety, the FDA has also released consumer alerts stating that no EV -based product is currently approved and cautioning against clinics that promote unapproved “stem cell” and EV interventions [53]. Given the intricate nature, heterogeneity, and incomplete elucidation of the mechanisms underlying EV products, the FDA aligns its regulatory oversight with the stringent requirements applicable to biologic drugs, including adherence to current Good Manufacturing Practice (cGMP) standards for quality control, safety testing, and consistency in manufacturing [15]. This rigorous regulatory stance reflects the agency’s commitment to balancing innovative advancements in regenerative medicine with patient safety, emphasizing the critical need for robust clinical evidence and standardized production processes before marketing authorization can be granted [52].

In summary, although EV therapies hold immense promise, the regulatory framework is still evolving. There is a clear need for adaptive and expansive guidelines to facilitate the safe transition of these therapies from the research phase to clinical practice, ensuring that their potential benefits are effectively realized [9,37].

### 3.2. European Union Regulatory Framework

The European Union (EU) predominantly classifies EV-based therapies as biological medicinal products, subjecting them to strict regulatory oversight similar to that applied to advanced therapy medicinal products (ATMPs) when their contents have a direct impact on physiological functions [15]. According to the European Directive (Directive 2001/83/EC) and Regulation 1394/2007/EC, EVs that are either directly purified from cells or contain functionally translated RNA with expected therapeutic effects are categorized as ATMPs. Consequently, these products are reviewed by the Committee for Advanced Therapies (CAT) at the European Medicines Agency (EMA), which assesses their quality, safety, and efficacy prior to granting marketing authorization [15]. Moreover, products that encapsulate recombinant nucleic acids or gene-modulating components may be regulated as gene therapy medicinal products under this framework [15].

The classification primarily depends on whether the EV composition exerts a specific mechanism of action affecting physiological functions, distinguishing them from ordinary biological specimens [15]. In addition, quality control, manufacturing processes, and clinical evaluations are required to meet Good Manufacturing Practices (GMP) and Chemical, Manufacturing, and Control (CMC) regulations, thus ensuring consistent pharmaceutical quality throughout the product lifecycle [15]. Nevertheless, the intrinsic heterogeneity and batch-to-batch variability of EV continue to pose significant challenges for standardization and regulatory harmonization [15].

Notably, the use of human-derived EV in cosmetic products is prohibited under EU Cosmetic Regulation (EC) No 1223/2009 due to safety concerns, such as risks of contamination and immunogenicity [54]. Therefore, regulatory pathways for EV-based therapies focus primarily on therapeutic applications rather than cosmetic uses [54].

Furthermore, the EMA actively issues scientific recommendations and guidelines to promote the safe clinical translation and commercialization of EV-based therapeutics. These recommendations emphasize the necessity for clear product characterization, a comprehensive mechanistic understanding, and rigorous clinical evaluation to safeguard patient safety while fostering innovation in this emerging field [15].

In summary, the EU regulatory framework mandates that EV therapies be managed as sophisticated biological medicinal products—often as ATMPs—with oversight by the EMA’s CAT committee, strict adherence to GMP, and comprehensive clinical evaluations to ensure safety and efficacy prior to market authorization. Although this framework accounts for the unique nature of EV, it also underscores ongoing challenges in quality control and standardization that will require continuous refinement [15,54].

### 3.3. Japan Regulatory Framework

In Japan, the regulatory framework for EV therapies is defined by a dual-track system designed to expedite the availability of regenerative medicinal products while ensuring patient safety. This system, influenced by the Pharmaceuticals, Medical Devices, and Other Therapeutic Products Act (PMD Act) and the Act on the Safety of Regenerative Medicine (ASRM) enacted in November 2014, promotes innovative therapies by allowing conditional and time-limited approvals for regenerative products, including EV therapies [47,55,56].

Nevertheless, Japan currently does not have specific legislation or detailed regulations that directly address EVs, such as EV, beyond the general oversight provided by the Medical Practitioners’ Act and the Medical Care Act [57]. Consequently, EV-based therapies are often utilized in clinical settings without comprehensive scientific validation or regulatory scrutiny, resulting in their widespread application even in the absence of robust efficacy or safety data. Moreover, the lack of mandated tracking and reporting mechanisms for adverse events further complicates patient safety oversight and emphasizes the urgent need for clearer regulations [58].

Although the dual-track system is intended to balance patient access with safety by employing risk-based classifications and certified review committees, the inherent variability of EV, stemming from differences in origin, culture conditions, and manufacturing methods, poses significant challenges to standardization and quality control that current cell therapy frameworks do not fully address [15]. Consequently, there is a growing call within the scientific and regulatory communities for dedicated guidelines and regulatory measures specifically tailored to EV therapies to ensure consistent product quality and patient protection while still promoting innovation [15].

In summary, while EV therapies in Japan are currently managed as biologic medicinal products under existing pharmaceutical laws, the absence of EV-specific legislation and comprehensive safety monitoring has led to regulatory ambiguity and potential patient risks. Therefore, enhanced regulatory oversight, clearer classification guidelines, and improved adverse event tracking are imperative to support the safe clinical development and application of EV-based therapies in Japan [15]

### 3.4. South Korea Regulatory Framework

South Korea’s oversight of EV therapies is primarily determined by the “Act on the Safety of and Support for Advanced Regenerative Medicine and Advanced Biological Products,” enacted in August 2019 and set to take effect in February 2025 [59]. This legislation is designed to safeguard patient safety and ensure the quality of advanced regenerative treatments, including EV-based therapies, by establishing stringent oversight over their development, manufacturing, and clinical application [15]. In this framework, both the Ministry of Food and Drug Safety (MFDS) and the National Institute of Food and Drug Safety Evaluation (NIFDS) play pivotal roles by issuing detailed guidelines that cover quality standards as well as nonclinical and clinical evaluation criteria specifically for EV therapeutics [15]. Moreover, EV products are classified as biologics and regulated under standards similar to those for cellular and gene therapies, although with tailored requirements that address their unique characteristics [15]. The MFDS enforces compliance with GMP specialized for advanced biopharmaceuticals, thereby emphasizing rigorous quality control and safety protocols to tackle challenges such as exosome heterogeneity and production variability [15]. Additionally, the regulatory framework facilitates patient access by allowing controlled clinical research pathways, which are authorized by designated institutions and overseen by a national review committee composed of scientific and medical experts [59]. Notably, South Korea’s approach distinguishes itself by excluding minimally manipulated cells, such as cord blood, from this advanced regenerative medicine pathway—a distinction that sets it apart from other international frameworks [59]. Furthermore, recent regulatory milestones, such as the MFDS’s authorization of S&E Bio’s Phase 1b clinical trial for an EV -based stroke therapy, underscore the advancements enabled by this landscape [60]. Collectively, South Korea’s regulatory structure embodies a comprehensive, science-driven approach that effectively balances the promotion of innovation with patient safety, thereby positioning the country as a leader in the global development and commercialization of EV therapeutics [15].

### 3.5. Taiwan Regulatory Framework

In Taiwan, EVs are recognized as cell-derived products within the realm of regenerative medicine, as explicitly defined by the recently enacted Regenerative Medicine Act (RMA) [47]. The RMA encompasses genes, cells, and their derivatives, thereby including EVs as regulated biological products. This systemic approach draws inspiration from regulatory models in Japan and South Korea while being tailored to Taiwan’s unique biomedical landscape [47]. Consequently, EV therapies are categorized as biologic medicinal products and must adhere to stringent quality and safety standards similar to those applicable to cell and gene therapies [15].

The primary regulatory authority overseeing EV therapy in Taiwan is the Taiwan Food and Drug Administration (TFDA), supported by the Center for Drug Evaluation [61]. The TFDA is charged with ensuring compliance with Good Tissue Practice (GTP), GMP, and Good Clinical Practice (GCP) standards throughout the entire lifecycle of EV products—from raw material procurement to clinical application [61]. Simultaneously, the CDE provides technical evaluations and detailed scientific assessments during the review of submission dossiers for both regenerative medical technologies and preparations, which include EV-based therapeutics [62].

Taiwan’s regulatory framework is dual-faceted, governing both regenerative medical technology—covering clinical use protocols—and regenerative medical preparations, which encompass biologic drug products such as engineered EVs [62,63]. For regenerative medical preparations, manufacturers must comply with site registration requirements and adhere to GMP standards, which may involve foreign inspections or Plant Master File (PMF) reviews, depending on the product’s country of origin [62]. Moreover, the development and manufacturing processes for EV therapies must overcome challenges related to their heterogeneity and instability by standardizing raw materials, controlling cultivation environments, optimizing purification procedures, and ensuring thorough characterization of their physicochemical and biological properties [15]. To this end, Taiwanese guidelines have incorporated international recommendations, such as the ISEV, Minimal Information for Studies of Extracellular Vesicles (MISEV) 2018, to standardize quality control and characterization practices [15].

EV therapies are subject to a phased approval process in which clinical trial applications must comply with the Human Trials Management Regulation, in alignment with international GCP standards [64]. Products that involve EV with minimal manipulation or systemic effects may qualify for expedited or conditional approval pathways, analogous to the “fast track” processes available for certain cell therapies in Taiwan [62]. However, a rigorous demonstration of pharmacokinetics, mechanisms of action, safety, and efficacy remains mandatory [15]. Additionally, the TFDA conducts post-marketing surveillance to track adverse events and treatment efficacy, which is critical due to the risks linked with biologics, including immunogenicity and off-target effects. All licensed institutions are required to report adverse drug reactions and submit annual summary reports. Furthermore, Taiwan’s proactive regulatory environment and supportive government initiatives have spurred local industry participation and clinical research in EV therapy. For instance, ExoOne Bio has obtained approval to use human-derived EVs as cosmetic ingredients following a rigorous review process conducted by the Ministry of Health and Welfare and the TFDA [65]. In summary, Taiwan’s regulatory framework for EV therapy reflects its commitment to advancing innovative medical treatments through a dual-framework approach that addresses significant scientific and regulatory challenges. As the field progresses, further regulatory adaptations will be essential to ensure the successful clinical implementation of EV-based therapies.

### 3.6. Chinese Regulatory Framework

EV therapy represents an emerging frontier in biomedical science in China, attracting significant attention and prompting the development of a specialized regulatory framework to manage both its research and commercialization. Since 2017, China has implemented a dual-track regulatory system that distinguishes between pathways for investigator-initiated studies and commercial clinical trials involving cell-based therapies, including EV products. This bifurcated system establishes clear and distinct requirements that facilitate the progression from research to market approval, thereby enhancing regulatory clarity and oversight for these innovative treatments [66].

At the core of this framework is the National Medical Products Administration (NMPA), which serves as the primary authority overseeing EV therapies, particularly those classified as cell therapy products. The NMPA is responsible for managing clinical trial approvals, enforcing quality control standards, and granting market authorization, all while ensuring that safety and efficacy criteria are rigorously evaluated before any therapeutic product reaches patients [66]. Complementing these measures, China has adopted GMP that conforms to international guidelines, such as PIC/S GMP. These standards are critical for ensuring quality assurance throughout the production and distribution stages, emphasizing standardized production environments, consistent product quality, and full traceability from raw materials to final products—factors that are essential in addressing the inherent heterogeneity of EV preparations [66].

Moreover, government policies play a pivotal role in bolstering the EV therapy sector in China. Strategic initiatives like the “Healthy China 2030” plan allocate substantial funding towards precision medicine and regenerative therapies, thereby indirectly accelerating innovation and the expansion of EV research and applications [67]. Additionally, regulatory efforts have yielded fast-track approvals for over 40 EV-based therapies as of 2023, highlighting a proactive stance aimed at expediting the availability of promising treatments while maintaining rigorous oversight [67]. Nonetheless, significant challenges remain. The complexity of EV’s molecular composition and their dynamic biological functions continues to impede full standardization. In response, China has adopted a cautious regulatory approach that requires comprehensive preclinical and clinical data to ensure reliable therapeutic outcomes, in line with international standards [66].

In conclusion, China’s regulatory framework for EV therapy is evolving in step with technological innovations and emerging regulatory demands. With its emphasis on safety, efficacy, and international alignment, the framework reflects a growing recognition of EV therapy as a key element in future therapeutic strategies [66,67].

### 3.7. Indian Regulatory Framework

In India, the principal regulatory body for the approval, clinical trial conduct, and commercialization of EV-based therapies is the Central Drugs Standard Control Organization (CDSCO), operating under the Ministry of Health and Family Welfare (MoHFW) [68]. The Drugs Controller General of India (DCGI), who heads CDSCO, serves as the Central Licensing Authority responsible for granting permissions related to clinical trials and marketing authorization for new drugs, including biological products such as EV therapeutics [69]. Accordingly, the CDSCO’s responsibilities include the approval of investigational new drugs, oversight of clinical trial protocols, and the enforcement of drug standards to ensure product safety and efficacy [68].

In addition, the Indian Council of Medical Research (ICMR) plays a pivotal role by issuing national guidelines focused on ethical conduct, research approval, and the monitoring of stem cell and related advanced therapies. Particularly, the ICMR’s 2017 guidelines mandate that stem cell interventions, including EV applications, must occur within sanctioned clinical trials. These trials require stringent oversight from Institutional Committees for Stem Cell Research (IC-SCR) and Institutional Ethics Committees (IEC) [70].

Although a dedicated regulatory framework exclusively for EV therapies has yet to be finalized, their governance in India currently falls under existing frameworks for biological products, stem cell research, and regenerative medicine as stipulated by the Drugs and Cosmetics Act, 1940, and its accompanying rules [71]. Under these regulations, EV-based products are treated as biological medicinal products that must comply with GMP and meet quality standards established by CDSCO [71]. Moreover, in 2019 the Government of India issued national guidelines on gene therapy product development and clinical trials, which, although primarily targeting gene therapy, provide procedural references applicable to advanced biotherapeutics and new pharmaceutical products, including EV [23] Consequently, clinical trials involving EV therapies must obtain prior approval from CDSCO (via the DCGI) and the respective registered Ethics Committees, adhering to the comprehensive provisions of the New Drugs and Clinical Trial Rules (2019). These rules require the detailed submission of data on pharmacokinetics, safety, immunogenicity, and manufacturing quality before initiating first-in-human trials [69]. Furthermore, CDSCO mandates the registration and continuous monitoring of Ethics Committees to ensure ongoing ethical oversight throughout clinical trials involving novel biologics and cell-based therapies. In addition, each trial site must secure approval from a registered Ethics Committee to provide multi-tiered review that safeguards participant rights and safety [69].

However, India’s current regulatory framework for EV therapy faces several challenges. Notably, the absence of dedicated, explicit guidelines that comprehensively address the unique characteristics of EV products, including considerations related to donor eligibility, purification standards, potency assays, and long-term safety monitoring, has resulted in regulatory ambiguity. This lack of a specific drug classification for EV products further complicates both clinical translation and commercial approvals [23].

Therefore, to ensure the safe, effective, and ethical use of EV therapies, the Indian regulatory system requires specific amendments and robust policy development. These should include the establishment of dedicated regulatory guidelines for the manufacture, quality control, clinical trials, and post-marketing surveillance tailored to the distinctive nature of EV products [23].

### 3.8. United Kingdom Regulatory Framework

The regulatory framework for EV therapy in the United Kingdom is notably stringent, reflecting the uncertainties and potential risks inherent to these emerging biological products. EV, broadly classified as EVs, are considered biological medicinal products and are consequently regulated as advanced therapy medicinal products (ATMPs) by the Medicines and Healthcare Products Regulatory Agency (MHRA) [72]. This status requires that their quality, safety, and efficacy undergo rigorous evaluation prior to market authorization, in accordance with both European Union and international standards for cell and gene therapies [73]. Currently, no EV-based therapies have received approval for clinical or cosmetic use within the UK, largely due to insufficient clinical trial data to substantiate their safety and effectiveness [74]. The MHRA has expressly stated that exosome injections, particularly those used for aesthetic purposes like facial rejuvenation, cannot be legally administered until robust evidence from controlled studies is provided [75]. As a result, clinics offering human-derived EV products, such as those obtained from umbilical cord blood or mesenchymal stem cells, are in violation of UK regulations, a situation that has sparked calls for stricter enforcement measures to protect public health [76]. Moreover, regulatory oversight includes strict adherence to GMP to ensure the purity, stability, and reproducibility of EV preparations, as well as robust quality control to mitigate risks such as microbial contamination or viral transmission [72]. UK authorities also follow guidance from organizations like the ISEV and the European Medicines Agency (EMA) for standardization and monitoring of these products [72]. Additionally, adverse events associated with unlicensed EV therapies are required to be reported under established pharmacovigilance frameworks, although existing surveillance systems for these novel interventions still exhibit certain gaps [52].

In summary, the UK’s regulatory framework for EV therapy is characterized by cautious and comprehensive control under the ATMP designation, emphasizing patient safety through stringent pre-market evaluation and the prohibition of unapproved clinical applications. This careful approach, while limiting immediate availability, is designed to prevent public exposure to unproven and potentially hazardous treatments until further scientific validation is achieved [74].

### 3.9. Switzerland Regulatory Framework

In Switzerland, EV therapy is primarily regulated by Swissmedic, the Swiss Agency for Therapeutic Products, which is responsible for authorizing and supervising therapeutic products to ensure their quality, safety, and efficacy [77]. EV-based products are classified as advanced therapy medicinal products (ATMPs) in accordance with European Union Regulation (EC) No 1394/2007, a framework with which Switzerland formally aligns to promote regulatory harmonization (© Copyright Swissmedic 2019, n.d.) [78]. This alignment subjects EV therapies to stringent oversight comparable to that applied to other cellular and gene therapies, employing a risk-based, case-by-case evaluation that accounts for the products’ biological complexity [77]. Moreover, the production, characterization, and quality control of these therapies adhere to international standards, such as those established by the ISEV, with particular emphasis on achieving the purity, potency, and reproducibility expected of clinical-grade EV [72]. Although specific authorizations for EV products by major regulatory bodies like the FDA remain absent, ongoing global clinical trials highlight both the promise and the regulatory challenges of EV therapies—a development that Swissmedic actively monitors within its evolving framework [72].

In conclusion, Switzerland’s regulatory environment for EV therapy is marked by rigorous evaluation aligned with advanced therapy standards, prioritizing patient safety and product quality while fostering convergence with European Union regulations to support innovation in regenerative medicine [77].

## 4. Regulatory Challenges in Clinical Trials and EV Research

The rapid evolution of EV research has been accompanied by significant regulatory challenges that substantially affect their clinical use as therapeutic agents. One of the primary issues is the appropriate classification of EV within existing regulatory frameworks. Regulatory agencies—most notably the U.S. FDA—are still determining whether EV should be regarded as biological products, which would subject them to stringent biomanufacturing standards, or as drug delivery systems that might follow alternative approval routes [9,37]. Furthermore, a core regulatory challenge arises from the absence of standardized methodologies for exosome isolation, purification, and characterization. This methodological variability can lead to inconsistencies in EV potency and therapeutic efficacy, thereby complicating compliance with regulatory requirements [2,79,80]. Without established protocols to guarantee the purity and precise content of EV preparations, concerns regarding patient safety and treatment effectiveness persist. In addition, regulatory bodies insist on a clear demonstration of the pharmacokinetics and biological activity of EV therapies; however, the complex nature of these vesicles renders such assessments particularly challenging [81,82].

Moreover, the scalability of EV production remains unresolved. Current production techniques often fail to generate EV in commercially viable quantities while maintaining consistent quality [5,80,83]. The requirement to adhere to GMP further complicates production, as many academic institutions and early-stage companies may not have the necessary infrastructure to meet these standards [80,84]. Consequently, the transition from preclinical investigations to clinical applications critically depends on the development of robust production and quality assurance protocols that satisfy regulatory expectations [38,80,85]. Additionally, the intrinsic variability in EV composition—stemming from differences in cellular origin, cell passage number, culture mediums, and environmental conditions—challenges conventional diagnostic and therapeutic evaluations and underscores the need for specific regulatory guidelines tailored to EV products [15].

Furthermore, an incomplete understanding of the physiological mechanisms underlying EV therapeutic effects, particularly regarding their pharmacokinetics, cellular uptake, and mechanism of action (MOA), impedes the establishment of reliable potency assays and comprehensive safety profiles. This lack of mechanistic clarity further complicates regulatory approval and the assurance of consistent therapeutic outcomes [2,84]. Thus, rigorous validation and standardization of EV products are essential for advancing this field [5,37,38].

In summary, navigating the regulatory landscape for EV-based therapies requires the development of standardized protocols for isolation and characterization, scalable manufacturing processes, and comprehensive regulatory compliance. Addressing these multifaceted challenges is critical to unlocking the clinical potential of EV therapeutics [15,86,87].

**Figure 2:**
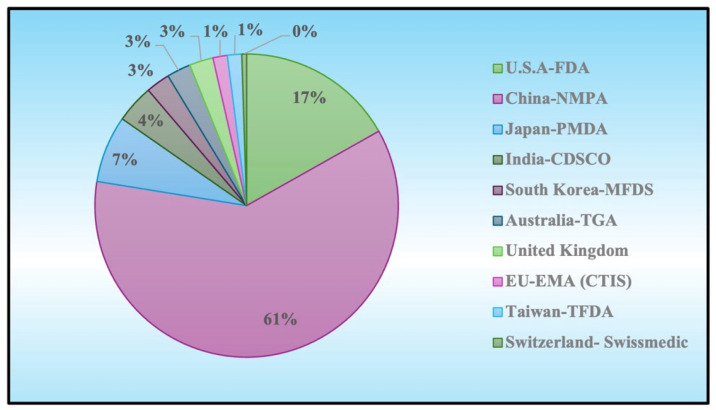
The proportion of EV-based clinical trials registered in the WHO ICTRP by regulatory jurisdiction. China-NMPA accounts for 61% of total registrations, followed by the U.S.A.’s FDA (17%), Japan’s PMDA (7%), India’s CDSCO (4%), South Korea’s MFDS, Australia’s TGA, and the United Kingdom (3% each), with smaller shares from the EU’s EMA (CTIS) (1%), Taiwan’s TFDA (1%), and Switzerland’s Swissmedic (<1%).

To assemble a comprehensive overview of current and ongoing EV-based clinical trials, we interrogated the World Health Organization’s International Clinical Trials Registry Platform (ICTRP) using the keywords “Exosome” and “Extracellular Vesicle,” Figure 2, thereby capturing data from the regions summarized in Table 2. The resulting dataset confirms that EV-based investigational products are regulated globally under established biologics or advanced-therapy frameworks. Specifically, in the United States, EV therapeutics require submission of an Investigational New Drug (IND) application to FDA’s CBER or CDER under 21 CFR Part 312 [88]. In the European Union, the EMA classifies EVs as advanced therapy medicinal products (ATMPs) per Regulation (EC) No 1394/2007; sponsors file a single Clinical Trial Application (CTA) via the centralized Clinical Trials Information System under Regulation (EU) No 536/2014 and obtain Committee for Advanced Therapies (CAT) classification [89,90]. In Japan, EVs fall under the PMD Act as regenerative-medical products, necessitating a Clinical Trial Notification (CTN) to PMDA [91]. South Korea’s Ministry of Food and Drug Safety regulates EVs as biologics requiring IND-type authorization, IRB approval, and ICH-GCP compliance [92]. Taiwan’s TFDA classifies EVs as new biologics under the Human Research Act, mandating IND applications or CTN filings with centralized IRB cooperation [93]; India’s CDSCO governs EV trials under the New Drugs and Clinical Trials Rules (NDCTR 2019), requiring CTA submissions and ethics-committee review [94]. In Australia, EV research proceeds under the TGA’s CTN (notification) or CTX (full-review) schemes with Human Research Ethics Committee oversight [95], whereas China’s NMPA employs a dual-track IND system for biologics and cell therapies [96]. Switzerland administers EV trials via Swissmedic under the Therapeutic Products Act and Human Research Act using ClinO-compatible CTA dossiers [97], and the United Kingdom regulates EV therapeutics as ATMPs under the Medicines for Human Use (Clinical Trials) Regulations 2004, with combined MHRA and Research Ethics Committee review through IRAS [98]. Across all jurisdictions, adherence to core ICH guidelines—such as Q5A/Q5B for quality and E6 for good clinical practice—provides a consistent foundation for trial design and conduct.

## 5. Harmonization of the EV Regulatory Framework

**Figure 3:**
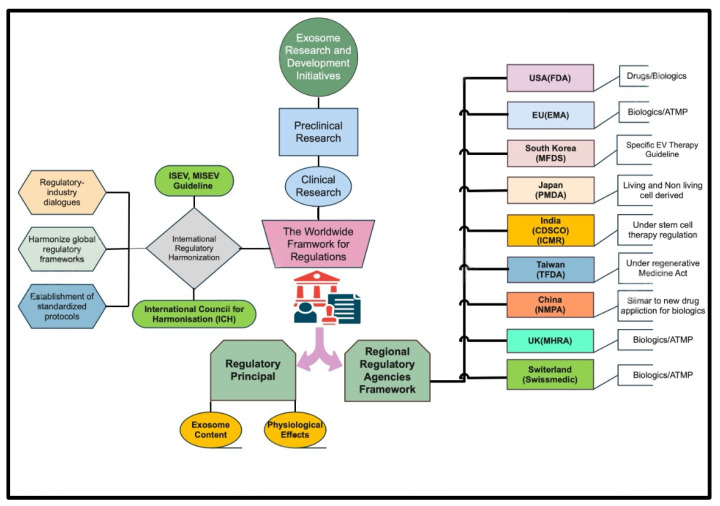
**Global regulatory ecosystem for EV-based therapeutics.** Centralized ICH-driven harmonization through ISEV/MISEV guidelines informs regional frameworks, guiding content characterization and safety assessment. Divergent country-specific pathways—USA (FDA), EU (EMA), South Korea (MFDS), Japan (PMDA), India (CDSCO/ICMR), Taiwan (TFDA), China (NMPA), UK (MHRA), Switzerland (Swissmedic)—are mapped alongside regulatory classifications (e.g., biologics, ATMPs) and approval processes. This schematic underscores the complexity and the need for unified standards.

International harmonization of the EV regulatory framework presents both a significant challenge and a promising opportunity in biomedicine. The inherent complexity of EV biology means that their development and manufacturing processes encounter unique regulatory obstacles. Globally, differing regulatory approaches result in variations in how EVs are characterized and evaluated for clinical use. Wang and colleagues underscore that achieving harmonization across these practices is crucial for ensuring safety and efficacy across diverse jurisdictions Table 1 [15]. One promising strategy to advance harmonization is the establishment of international cooperation frameworks. Current discussions advocate a dual approach: one that focuses on the composition and biological effects of EV, and another that evaluates their therapeutic implications [15]. In support of this, the ISEV has called for the standardization of methodologies to assess exosome products, thereby promoting a unified regulatory approach that spans both geographical and institutional boundaries [45] Figure 3. Such collaborative efforts are essential, as inconsistent regulations can impede innovation and delay the translation of research into clinical applications. Moreover, international bodies such as the International Conference on Harmonization (ICH) play a pivotal role in aligning regulatory standards for biopharmaceuticals, including EV. Their established frameworks have fostered regulatory convergence across regions, facilitating smoother collaborative efforts in drug development and approval processes [99]. Additionally, recent initiatives emphasize sharing best practices and aligning preclinical and clinical evaluation standards to further bolster regulatory coherence in EV therapeutics [100,101]. Despite these advancements, notable challenges remain. For example, incomplete harmonization—illustrated by experiences in the stem cell sector—can lead to regulatory fragmentation, thereby compromising global assessments of efficacy and safety [102]. Furthermore, stakeholders must navigate complex ethical, legal, and social considerations as they work toward a more cohesive regulatory stance on EV [103]. Addressing these multifaceted issues necessitates an ongoing dialogue among regulatory authorities, scientists, and healthcare providers to develop robust standards that support innovation while safeguarding public health Figure 3.

In conclusion, progress toward harmonizing the regulatory framework for EV therapies is a multifaceted journey marked by both significant achievements and persistent challenges. Continued dialogue and alignment among global regulatory agencies are essential for establishing mutually recognized standards, streamlining approval pathways and methodologies, enhancing regulatory efficiency, fostering trust, and ultimately improving patient access to these innovative therapies [15,45,99,100,101,102,103].

## 6. Future Directions, Opportunities, and Policy Implications

As recognition of EV’ therapeutic potential grows, their regulatory framework continues to evolve. Given the inherent complexity of EV biology and their diverse clinical applications, it is imperative to develop comprehensive regulatory guidelines that ensure both safety and efficacy. Currently, existing regulations often lack the necessary specificity regarding EVs, thereby complicating their development and approval. Accordingly, efforts should concentrate on clearly defining EV within regulatory frameworks—whether as biological products or drug delivery systems—especially in light of their complex biogenesis and biocompatibility [15,37,52]. Such clarity will enable researchers and companies to navigate the regulatory landscape more effectively.

Moreover, advancements in exosome isolation and characterization techniques are expected to play a crucial role in future research and development. The improvement and standardization of these methods will enhance the reproducibility and purity of exosome preparations, thereby facilitating more reliable clinical applications [2,104]. In addition, integrating artificial intelligence and machine learning into data analysis could streamline the biomarker identification process, thus accelerating the translation from basic research to clinical trials [105].

EVs also present novel therapeutic opportunities, particularly in the field of personalized medicine. Their capacity to transport nucleic acids, proteins, and lipids specific to the originating cell allows for targeted therapies in diseases such as cancer, neurodegenerative disorders, and metabolic conditions [38,106]. Furthermore, EV -mediated drug delivery systems offer the potential to improve treatment efficiency while mitigating the adverse effects commonly associated with conventional therapies [107]. Global collaboration is another vital avenue for optimizing both regulatory and investigational processes in EV research. Initiatives by international bodies such as the ISEV are essential for fostering a unified approach to developing and implementing EV therapies [108]. Harmonizing regulatory standards across countries could facilitate a smoother transition of EV-based products into clinical practice, thereby accelerating medical innovation.

Given the rapid advancements in this field, regulatory frameworks must adapt accordingly. Policymakers need to ensure that regulations are both comprehensive and flexible enough to accommodate ongoing scientific and technological progress. This includes recognizing EVs as pivotal components of biologics, which necessitates stricter guidelines concerning their composition, manufacturing, and clinical applications [109]. Additionally, regulatory bodies should consider establishing fast-track pathways for promising EV therapies similar to accelerated approval processes used for other innovative treatments, to expedite patient access while maintaining rigorous safety and efficacy standards [110]. Complementary policy efforts should also focus on incentivizing research and development in EV technology through targeted grants and funding, thereby empowering emerging scientists and organizations to contribute meaningfully to this field [111,112].

## 7. Conclusions

In summary, EV-based therapies hold substantial promise, driven by ongoing scientific advances and an evolving regulatory environment. Nevertheless, the global regulatory landscape is filled with significant challenges, including the absence of harmonized international guidelines, technical obstacles in characterization and manufacturing, and unresolved concerns regarding safety and efficacy. Consequently, the development of clear and robust policies is expected to streamline the advancement process and promote collaboration among researchers, clinicians, and industry stakeholders, ultimately enabling safe and effective applications across diverse medical fields. By tackling regulatory voids and adopting technological developments, EV therapeutics can foster improved patient care.

## Figures and Tables

**Table 1:** Overview of regional regulatory frameworks for EV-based therapeutics, detailing the responsible authorities, classification status, pathways, and principal evaluation criteria across key jurisdictions.

Region/Country	Regulatory Authority	Regulatory Status & Classification	Classification Focus
United States	U.S. Food and Drug Administration (FDA)	EV are regulated as biologics/drugs and subject to premarket review; no products approved to date	Content characterization; physiological function
European Union	European Medicines Agency (EMA)	EV fall under the advanced therapy medicinal product (ATMP) regulation; classification criteria remain unclear	Cargo composition; functional (RNA) content
Japan	Pharmaceuticals and Medical Devices Agency (PMDA)	Dedicated subcommittees evaluate safety and quality of EV therapies	Source of manufacture; living vs. nonliving
South Korea	Ministry of Food and Drug Safety (MFDS)	Published specific guidelines for EV–based therapies	Manufacturing source
Taiwan	Taiwan Food and Drug Administration (TFDA)	Regenerative Medicine Development Act encompasses EV; cosmetic use explicitly permitted	Manufacturing source; regenerative applications
India	Central Drugs Standard Control Organization (CDSCO) & ICMR	Stem-cell therapies regulated; no EV-specific therapeutic guidelines established	Nascent and evolving
Australia	Therapeutic Goods Administration (TGA)	Stem-cell and tissue therapies regulated since 2019; no dedicated EV guidelines	Nascent and evolving
China	National Medical Products Administration (NMPA)	EV products regulated under the same framework as biological new drug applications	Nascent and evolving
Switzerland	Swiss Agency for Therapeutic Products (Swissmedic)	EV-derived products classified as biological medicines; may be regulated as ATMPs when cells are extensively manipulated	Nascent and evolving
United Kingdom	Medicines and Healthcare Products Regulatory Agency (MHRA)	EV therapies classified as biological medicinal products; ATMP framework applies if derived from manipulated cells	Nascent and evolving

**Table 2:** Regional distribution of EV-based clinical trials registered in the WHO ICTRP, showing trial counts by jurisdiction, product classification and corresponding regulatory submission pathways.

International Clinical Trials Registry Platform (ICTRP) by Region	Trial Counts	Classification	Regulatory Submission Pathways
U.S.A-FDA	33	Biologics (IND-regulated)	IND (21 CFR 312)
EU-EMA (CTIS)	3	ATMP (Reg EC 1394/2007)	CTA via CTIS (Reg EU 536/2014)
Japan-PMDA	14	Regenerative-medical product (PMD Act)	CTN (Clinical Trial Notification)
South Korea-MFDS	5	Biologics	IND authorization (CTN/CTA equivalent)
Taiwan-TFDA	3	New drug/Biologics	IND application; c-IRB/CTN for MRCTs
India-CDSCO	8	Biologics/New drug (NDCTR 2019)	CTA under NDCT Rules; IRB
Australia-TGA	5	Biologicals	CTN or CTX scheme
China-NMPA	119	Biologics/Cell-therapy dual track	IND submission (Pharma Admin Law)
Switzerland-Swissmedic	1	Medicinal products (ATMP-like under TPA/HRA)	CTA under ClinO; eDok_KLV dossier structure
United Kingdom	5	ATMP (MHCTR 2004/amend 2024)	CTA via IRAS combined MHRA/REC review

## Data Availability

All data are contained within the article.

## Abbreviations

The following abbreviations are used in this manuscript:

ATMP	Advanced Therapy Medicinal Products
CBER	Center for Biologics Evaluation and Research
CDER	Center for Drug Evaluation and Research
ISEV	International Society for Extracellular
FDA	Food and Drug Administration
GMP	Good Manufacturing Practices
MSC	Mesenchymal Stem Cell
PMD	Pharmaceuticals and Medical Devices
ASRM	Act on the Safety of Regenerative Medicine
EMA	European Medicines Agency
PMDA	Pharmaceuticals and Medical Devices Agency
MFDS	Ministry of Food and Drug Safety
TFDA	Taiwan Food and Drug Administration
CDSCO	Central Drugs Standard Control Organization
ICMR	Indian Council of Medical Research
TGA	Therapeutic Goods Administration
NMPA	National Medical Products Administration
MHRA	Medicines and Healthcare Products Regulatory Agency
PHS	Public Health Service
IND	Investigational New Drug
BLA	Biologics License Application
CMC	Chemical, Manufacturing, and Control
CAT	Committee for Advanced Therapies
RMA	Regenerative Medicine Act
GTP	Good Tissue Practice
GCP	Good Clinical Practice
MISEV	Minimal Information for Studies of Extracellular Vesicles
EV	Extracellular Vesicles
MOA	Mechanism of Action
ICH	International Conference on Harmonization
CTN	Clinical Trial Notification
CTX	Clinical Trial Exemption
CTA	Clinical Trial Application
ICTRP	International Clinical Trials Registry Platform
